# Development and validation of an automated microfluidic perfusion platform for parallelized screening of compounds in vitro

**DOI:** 10.1111/bcpt.13940

**Published:** 2023-09-17

**Authors:** Francesca R. Brugnoli, Marion Holy, Marco Niello, Julian Maier, Marcus Hanreich, Mario Menzel, Matthias Haberler, Niklas Zulus, Thomas Pickl, Christa Ivanova, Lisa D. Muiznieks, Benjamin Garlan, Harald H. Sitte

**Affiliations:** ^1^ Elvesys ‐ Microfluidic Innovation Center Paris France; ^2^ Center for Physiology and Pharmacology, Institute of Pharmacology Medical University of Vienna Vienna Austria; ^3^ Höhere Technische Bundeslehr‐ und Versuchsanstalt Mödling (HTL Mödling) Mödling Austria; ^4^ Hourani Center for Applied Scientific Research Al‐Ahliyya Amman University Amman Jordan; ^5^ Center for Addiction Research and Science ‐ AddRess Medical University Vienna Vienna Austria

**Keywords:** automated screening, microfluidics, neurotransmitter, release assay, serotonin

## Abstract

Monoamine transporters are of great interest for their role in the physiological activity of the body and their link to mental and behavioural disorders. Currently, static well‐plate assays or manual perfusion systems are used to characterize the interaction of psychostimulants, antidepressants and drugs of abuse with the transporters but still suffer from significant drawbacks caused by lack of automation, for example, low reproducibility, non‐comparability of results. An automated microfluidic platform was developed to address the need for more standardized procedures for cell‐based assays. An automated system was used to control and drive the simultaneous perfusion of 12 channels on a microfluidic chip, establishing a more standardized protocol to perform release assays to study monoamine transporter‐mediated substrate efflux. D‐Amphetamine, GBR12909 (norepinephrine transporter) and *p*‐chloroamphetamine, paroxetine (serotonin transporter) were used as control compounds to validate the system. The platform was able to produce the expected releasing (D‐Amphetamine, *p*‐chloroamphetamine) or inhibiting (GBR12909, paroxetine) profiles for the two transporters. The reduction of manual operation and introduction of automated flow control enabled the implementation of stronger standardized protocols and the possibility of obtaining higher throughput by increasing parallelization.

## 1 INTRODUCTION

Neurotransmitter transporters (NTTs) are transmembrane proteins located in the close vicinity of the synaptic cleft and terminate signal transmission by the presynaptic reuptake of dopamine (DA), norepinephrine (NE) and serotonin (5‐hydroxytryptamine [5‐HT]). Monoamine NTTs have been named according to their cognate substrates: dopamine transporter (DAT), norepinephrine transporter (NET) and serotonin transporter (SERT) (solute carrier 6 [SLC6]). Monoamines play essential roles in many physiological activities such as psychomotor, cardiovascular and respiratory function, gastrointestinal control, sleep, hormone secretion, body temperature and pain.^1^ Importantly, changes in the homeostasis of extracellular monoamines therefore play important roles in pathophysiological malfunction and thus may lead to disease.^2^ It is well established that monoamine homeostasis is not only regulated by exocytosis but also via reverse transport via NTTs in a calcium‐independent manner.^3^, ^4^, ^5^, ^6^ Transporter‐mediated efflux can be induced by changes in the extracellular ionic composition and by psychostimulants such as amphetamines.^7^

Antidepressant drugs such as selective SERT inhibitors like fluoxetine or escitalopram and drugs of abuse such as cocaine, 3,4‐methylenedioxymethamphetamine (MDMA), methylenedioxypyrovalerone (MDPV) or mephedrone target DAT, NET and SERT.^7^, ^8^, ^9^, ^10^, ^11^ Since drugs of abuse are typically barred by drug legislation, rogue chemists try to circumvent these barriers and create compounds with slightly changed chemical structures in order to achieve new and thus unscheduled substances.^12^ Therefore, understanding how compounds interact with NTTs helps to provide important functional and toxicological information on these novel psychoactive substances (NPS).

Different ligand–transporter interactions exist and could be exploited to study the molecular mechanism of the proteins or for therapies for neuropsychiatric disorders or substance abuse. The interaction can be classified according to the preferred binding site (orthosteric or allosteric), the type of effect on the transporter (inhibitory or releasing properties) and the efficacy of the ligand to elicit its effect (full or partial efficacy).^13^, ^14^, ^15^, ^16^ Ligands that have an inhibitory effect lock the transporter in a conformational state that prevents the transport of a given neurotransmitter, while ligands promoting substrate efflux into the synaptic cleft have releasing properties. Lastly, it needs to be noted that ligands can have decreased releasing efficacy in comparison to standard ligands (i.e., partial releasers^17^, ^18^) or inhibitory activity at some transporters and releasing activity at other transporters (i.e., hybrid compounds).^10^, ^19^

Various methods have been utilized to study molecular interaction between drugs and transporters. Radioligand or fluorescent‐based uptake assays were used alone^20^, ^21^ or in combination^22^ to study substrate transport. Mass spectrometry‐based assays can also be used to quantify the transport of substrates by transporters.^23^ In addition, electrophysiological approaches such as the patch‐clamp technique in the whole‐cell mode^24^ or the two‐electrode voltage‐clamp method^25^, ^26^ can uncover transport mechanisms by studying the electrical activity of transporters within the plasma membrane of cellular systems. The reverse operation of NTTs, however, is best studied by release assays.^21^

Release assays have been used to study transporter‐mediated efflux in vitro and ex vivo for different transporters.^27^, ^28^, ^29^ The method consists of loading cells or synaptosomes with radioactive substrate and then exposing them to the compound of interest.^19^, ^21^, ^30^, ^31^, ^32^, ^33^, ^34^, ^35^ Transfected cell lines have become popular because they overcome the limitations of ex vivo preparations, that is, they do not contain the entire vesicular machinery, and thus, results are not confounded by quantal release.^30^, ^31^ Moreover, cell lines express only the transporter of interest at a high density to allow their exclusive examination. Initially, they were placed in wells either adherent or in solution, preloaded with a radioactive substrate of the transporters. Then, cells are exposed to different concentrations of compounds of interest. Subsequently, the amount of released tritiated substrate over a time period is measured.^34^, ^36^, ^37^, ^38^ However, the static conditions of this method pose a risk of producing uncontrolled effects^39^; for instance, ongoing reuptake may occur. A possible solution to overcome some of the disadvantages of static methods is to rely on superfusion systems. A previously established perfusion system for release assays implements the use of perfused chambers under dynamic conditions,^29^, ^30^, ^40^, ^41^, ^42^, ^43^ thereby avoiding the ongoing reuptake of substrate via continuous flow. Yet, the superfusion instrument lacks flow control, uses manual valves, and requires an operator to perform actions during the course of the assay, introducing the possibility of human error during the experiments. This system also uses a large quantity of the compounds under scrutiny, which is a particular disadvantage when performing experiments with illicit drugs that may only be available in minute amounts.

Microfluidics is being widely employed in biological science and could help tackle these problems. It is based on the manipulation of fluids at the microscale. The small geometries and the high surface to volume ratio (SVR) create laminar flow conditions where diffusion is the primary process of heat and mass transport. This very controlled environment is key to the well‐known advantages of microfluidics such as low consumption of reagents, suitability for automation and parallelization and the ability to fine‐tune more parameters to customize the environment for a specific application compared to static methods.^44^, ^45^ To that effect, microfluidic systems have been developed to study the release of NTTs in single‐cell assays,^46^ neuronal cells,^47^ also adopting electrochemical detection.^48^ Nonetheless, most of the microfluidic systems currently being developed by research groups produce in‐house components (i.e., polydimethylsiloxane chips) and overall, there is no standardized procedure for cell‐based assays. To address these points, we developed an automated microfluidics‐based platform, added an automated collector unit and tested it with human embryonic kidney (HEK293) cells expressing NTTs. The system was successfully validated by obtaining the expected release profiles of compounds with known properties and can be further used to study previously uncharacterized illicit compounds.

## 2 MATERIALS AND METHODS

The study was conducted in accordance with the Basic & Clinical Pharmacology & Toxicology policy for experimental and clinical studies.^49^

### 2.1 Reagents and chemicals

D‐Amphetamine (D‐Amph) was sourced from Tocris (UK), paroxetine from Santa Cruz (USA), p‐chloroamphetamine (pCA) and GBR12909 were from Sigma‐Aldrich (USA). Hellmanex was from Hellma France S.A.R.L. (Paris, France). Radiochemicals are [^3^H] 1‐methyl‐4‐phenylpyridinium ([^3^H]MPP+; 80–85 μCi × mmol^−1^) from American Radiolabeled Chemicals (St. Louis, MO, USA) and [^3^H]5‐HT (28.3 μCi x mmol^−1^) from PerkinElmer (Boston, MA, USA). Other chemicals and cell culture supplies were obtained from Sigma‐Aldrich (St. Louis, MO, USA), and cell culture dishes were from Sarstedt (Nuembrecht, Germany).

### 2.2 Cell culture

Stable monoclonal HEK293 cell lines, overexpressing human NET and SERT, were established as previously described.^21^, ^35^ Cells were kept in culture in high glucose (4.5 g × L^−1^) and L‐glutamine‐containing (584 mg × L^−1^) Dulbecco's Modified Eagle Medium with 10% heat‐inactivated fetal bovine serum, penicillin (100 U × 100 mL^−1^) and streptomycin (100 mg × 100 mL^−1^). Selection was achieved by geneticin (G418) (50 mg × mL^−1^). Cells were usually grown in 10‐cm cell culture dishes in an incubator at 37°C and 5% CO_2_. The day prior to the assay, cells were seeded in a perfusion chamber of an IBIDI microfluidic chip (μ‐Slide VI 0.5 Glass Bottom, ibidi GmbH). Chambers were previously coated with poly‐D‐lysine for 20 min at 37°C and then cells were seeded at a density of approximately 1 · 10^6^ cells × mL^−1^.

### 2.3 Assay for measuring release of radiolabeled substrate in transfected HEK293 cells

The human NET (hNET) and human SERT (hSERT) employed in this work are part of the SLC6 family. The efflux of these membrane proteins is dependent on sodium thus, it can be selectively augmented by the addition of monensin (MON).^50^ MON is an ionophore that disrupts the pre‐existing sodium gradient of the cell, creating an increase of sodium inside the cell membrane and an outflow of protons. The use of MON in the release assay helps to identify the type of interaction between transporter and substance, that is, inhibitory or releasing. For the former, the effects are unaltered even in the presence of MON, while for the latter, there will be increased efflux in the presence of the ionophore. Transfected HEK293 cells were loaded with radioactive substrate (0.05 μM [^3^H]MPP+ (NET) or 0.08 μM [^3^H]5‐HT (SERT) for 20 min at 37°C) and then perfused with different solutions at a constant flow rate (0.6 μL·min^−1^). The assay was carried out modified from previous studies.^18^, ^50^ Briefly, the assay included washout with Krebs–HEPES buffer (KHB; 25 mM HEPES, 120 mM NaCl, 5 mM KCl, 1.2 mM CaCl_2_, 1.2 mM MgSO_4_, and 5 mM Glucose at pH 7.3; 20 min) to establish a stable baseline efflux, perfusion of cells with MON or vehicle (KHB) (10 μM; 6 min), addition of compounds of interest (D‐Amph 10 μM, GBR12909 10 μM, pCA 3 μM, paroxetine 0.05 μM; 10 min) to KHB with or without MON. At the end of the experiment, the cells were flushed using sodium dodecyl sulphate (SDS) to retrieve the remaining radioactivity. The interaction of detergent with the cells lysed the cell membranes and all the radioactive substrate was collected at the outlet. The supernatant was collected in 2 min fractions by an automated collector. The development of the automated collector is described in the following sections. Radioactivity was quantified with a beta‐scintillation counter (PerkinElmer, Waltham, MA, USA). The released amount of tritiated substrate for each fraction was expressed as the amount of released tritium compared to the amount remaining in the cell at the beginning of the pertinent fraction.

### 2.4 Data and statistical analysis

All data stem from at least three separate experiments (*n* = 3), in triplicate and are reported as mean ± standard deviation (SD). Data from release assays, in the presence of MON or vehicle over time, was statistically analysed using a two‐way ANOVA test. One‐way ANOVA was adopted to verify the homogeneity of the flow rate in all 12 channels using Tukey's multiple comparisons test.

## 3 RESULTS

### 3.1 Development of an automated microfluidic perfusion platform to standardize a procedure for cell‐based assays

This work focused on the development and characterization of a microfluidic system for pharmacological characterization of compounds. This system was designed to address the main challenges of continuous high‐precision flow control and automation to improve temporal and physiological relevance, reproducibility and interoperability and reduce the volume of chemicals required. The platform consisted of a perfusion module and an automated fraction collector.

**FIGURE 1:**
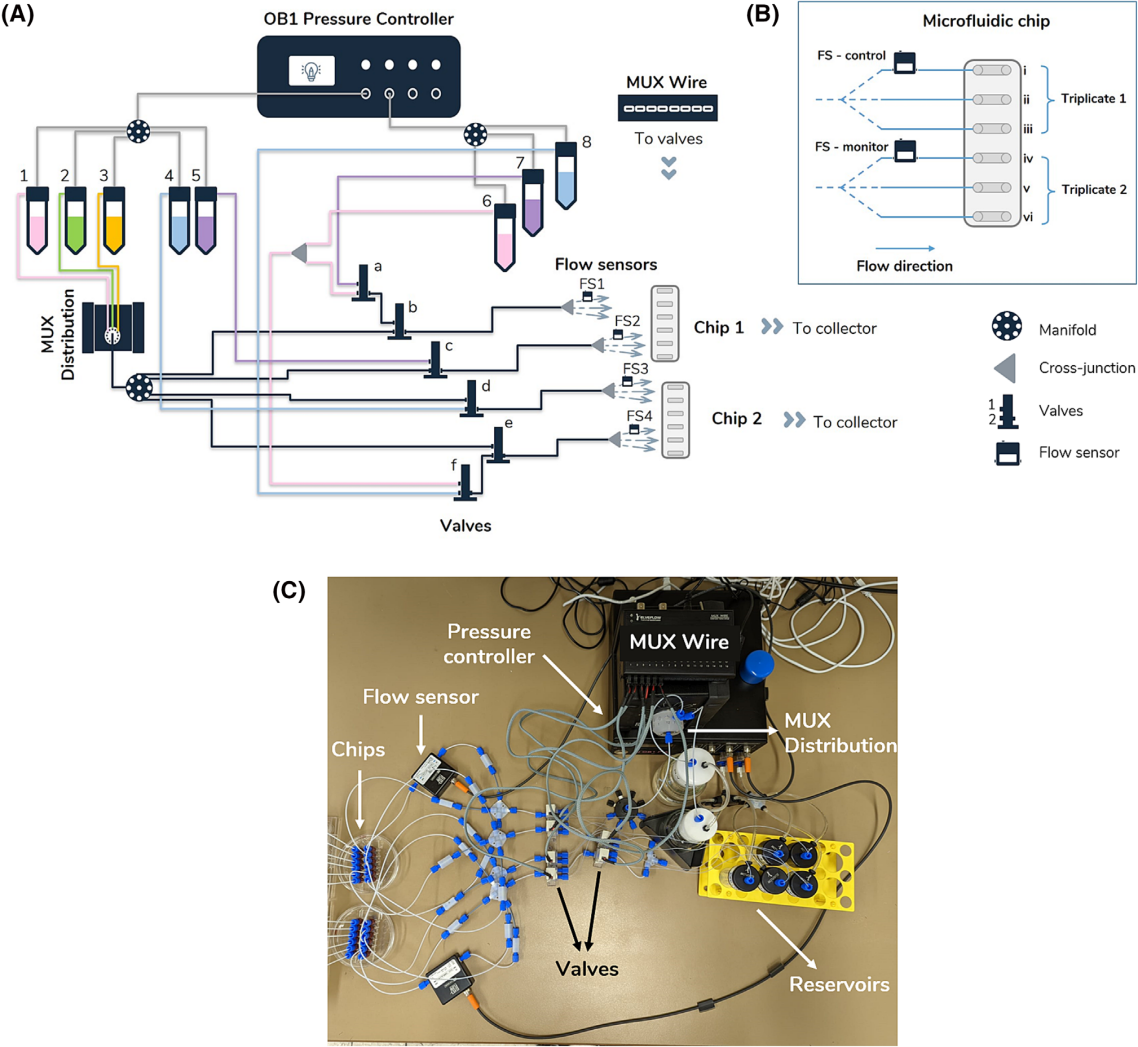
Microfluidic perfusion platform for automated release assay. (A) Schematic diagram of the perfusion module. A series of valves (rotary distribution and 3‐way) were used to control the flow of different solutions through 12 channels simultaneously (two chips × six channels each) as well as the sequential injection of solutions following a scheduled sequence (i.e., sequence followed injection of Krebs–HEPES buffer [KHB; buffer, Solution 1, 6] through all 12 channels simultaneously, then selected channels received monensin or buffer [Solutions 2, 6, respectively], followed by a releaser ± monensin [MON] [Solutions 5, 7], or an inhibitor ± MON [Solutions 4, 8], then sodium dodecyl sulphate [SDS] [Solution 3] in all 12 channels). (B) Schematic of chip (ibidi μ‐Slide III 3D perfusion) and connected flow sensors. (C) Assembled microfluidic perfusion module.

The perfusion module featured a pressure‐driven flow controller (OB1 MK3+, four pressure outlets × 2 bar; Elveflow, France) connected to a compressed air source to move liquid through the system with high precision, a rotary valve (12/1 MUX distribution) to sequentially inject reagents, eight 3/2 valves (microfluidic 3‐way valves with 2‐inlets/1‐outlet) to control the liquid pathway and four flow sensors to control or just monitor the flow rate (Figure 1).

This setup was designed to simultaneously perfuse two chips, each with six separate channels. Individual chips were used to test the effect of a different transporter‐compound pair, where this fluidic configuration enabled each chip to run triplicates of compound ± a reagent (i.e., in this case, MON). The assay consisted of a 20‐min washout to obtain a stable basal efflux, during which Solution 1 was directed through Valves *c* and *d*, into Triplicate 1, Chip 1 (Channels i, ii, iii) and Triplicate 2, Chip 2 (Channels x, xi, xii) (Figure 1B). At the same time, Solution 6 was directed through valves *a*, *b*, and *f*, *e* into Triplicate 2, Chip 1 (Channels iv, v, vi) and Triplicate 1, Chip 2 (Channels vii, viii, ix) (Figure 1B).

The addition of the ionophore MON can discriminate releasing agents from inhibitors by enhancing the efflux in the presence of a releasing agent.^51^ Thus, the microfluidic system was configured to perfuse one triplicate of each chip with MON (10 mM, Solution 2), and the other triplicate with KHB (Solution 6). For this step, a MUX distribution was used to sequentially select and inject MON (Solution 2), into Channels i–iii and Channels x–xii. The position of Valves *a*, *b*, *e* and *f* remained unchanged in order to maintain perfusion of KHB (Solution 6) in the remaining channels.

Following MON, compounds were added to benchmark the suitability of our microfluidic system for demonstrating a releasing efflux profile from hNET and hSERT. One releasing compound (D‐Amph, hNET or pCA, hSERT) and one inhibitory compound (GBR12909, hNET or paroxetine, hSERT) were tested per experimental approach. For hNET, D‐Amph + MON (Solution 5) was added by the activation of Valve *c* (i.e., after valve position change; into Channels i–iii), and D‐Amph only (Solution 7) was pushed through Valve *a* (Channels iv–vi). Concurrently, GBR12909 + MON (Solution 4) and GBR12909 only (Solution 8) were pushed through Valves *d* (into Channels vii–ix) and *f* (Channels x–xii), respectively. Lastly, all channels were perfused with SDS (Solution 3). The procedure was repeated for hSERT using compounds *p*CA and paroxetine.

The release assay was automated using a sequence scheduler integrated into the control software. Valves were scheduled to sequentially adopt different positions to automate the flow path and liquid injection according to the protocol (Table 1). Reservoir pressurization and flow rate control were also automated using the software.

**TABLE 1:** Valve configuration sequence to automate the release assay protocol. 3/2 valves were scheduled to sequentially adopt one of two positions, enabling the selection of two different flow paths. The MUX distribution valve can adopt one of 12 positions, facilitating the selection of up to 12 different liquid reservoirs (three valve positions were scheduled for this assay as three reservoirs were connected). Valve configuration was maintained for the indicated perfusion time of injected solutions.

	3/2 valves	MUX distribution valve	Injected solutions	Perfusion time (min)
Step 1 Washout	Position 1: b, d, f Position 2: a, c, e	Position 1	1, 6	20
Step 2 ±MON	Position 1: b, d, f Position 2: a, c, e	Position 2	2, 6	6
Step 3 Compound	Position 1: a, b, c Position 2: d, e, f	Position 2^a^	5, 7, 4, 8	10
Step 4 Lysis	Position 1: d, e, (a^a^) Position 2: b, c, (f^a^)	Position 3	3	4

^a^ Fluid bypassed the MUX distribution valve in Step 3, and 3/2 valves a, f in Step 4.

**FIGURE 2:**
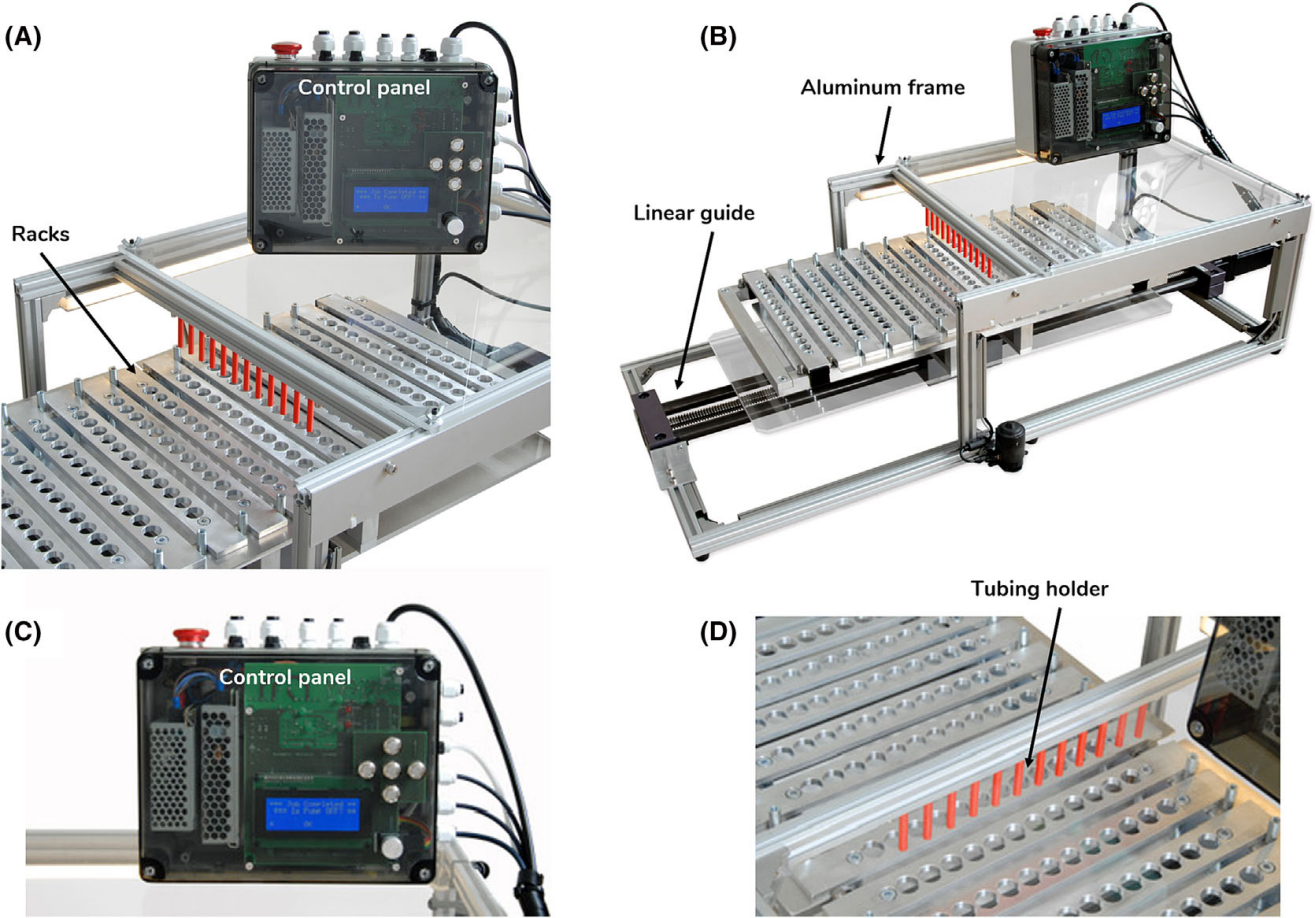
Automated fraction collector. (A) Side view of the racks and tubing outlets holder. Front view of control panel. (B) Side view of the entire device. Linear guide and aluminium frame. (C) Front view of control panel. (D) 3D printed tubing outlets holder.

A semi‐automated fraction collector was developed to satisfy the needs of the release assay to collect 12 fractions simultaneously at user‐defined time intervals. The collector featured 12 vertical outlet tubes fixed over a motorized base that advances rows in sets of 12 vials, in timed intervals (Figure 2). The specially developed and programmed circuit board creates an interface that nevertheless allows a wide range of settings. After the process sequence has been entered, the collector moves precisely to the vials, carries out washing processes and informs the operator visually and acoustically at which point the experiment is. Furthermore, the implementation of a loading and unloading function at the beginning and end of the process makes filling the carriage with the sample vials particularly easy. The vials to be filled are inserted into a specially developed rack. The rack‐holder can accommodate 14 racks in a modular fashion. Thereby, the racks can be changed in groups which improves the workflow, as six racks can be exchanged at the same time. The collector is based on an aluminium profile frame which offers stability. A linear guide is installed on this frame which ensures that the position is always accurate via a stepper motor and two inductive sensors. In order to keep the hoses in position and still allow for easy replacement, a 3D‐printed solution was developed. This contains a light cone which allows quick insertion and removal. The holder for the cells is also easy to exchange. On the upper plate of the collector, a system for attaching valves and the cell‐containing chips can be mounted, which can be completely moved and interchanged. Due to this implementation, the tubing of the collector is arranged in the simplest manner and as short as possible.

### 3.2 Characterization of perfusion parameters—flow rate and pressure

**FIGURE 3:**
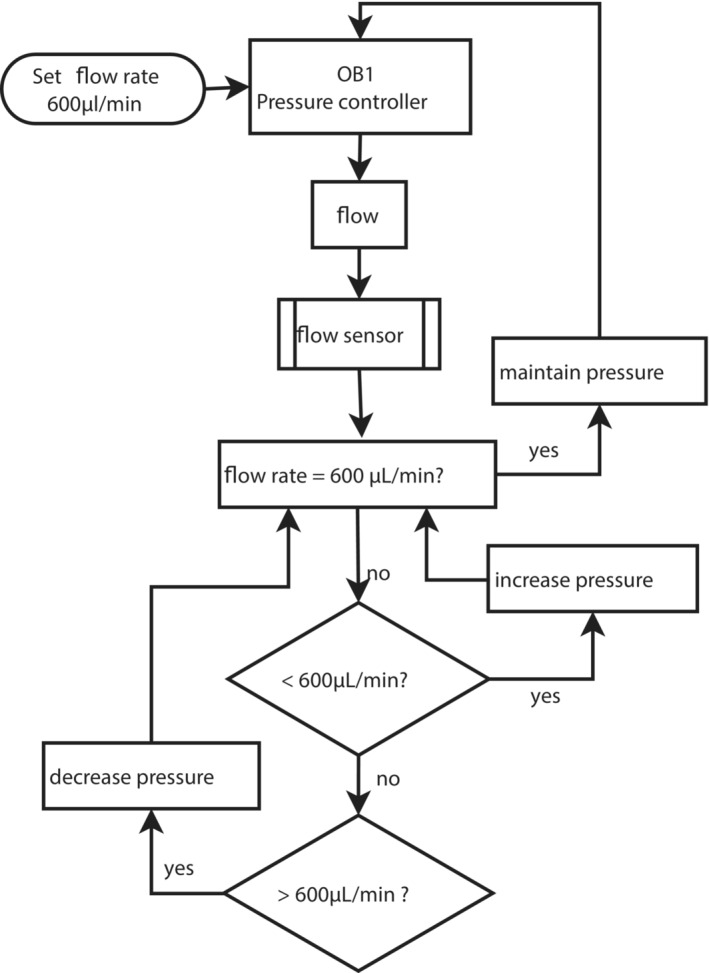
Flow chart of flow rate feedback control loop. Flow rate is set by the user in the software and continuously monitored by the flow sensor. A proportional–integral–derivative (PID) feedback control loop automatically computes the deviation between the set flow rate and the monitored flow rate and modifies the pressure in order to maintain the set rate. The response behaviour of the PID controller is defined by its three control variables (P, I, and D), whose values can be tuned for responsive and stable feedback control.

Precise manipulation of fluids through a complex network of tubing can be achieved by controlling flow rate and/or pressure. Control via flow rate requires the inline connection of flow sensors, but only one flow sensor can be paired per pressure outlet for feedback control. Thus here, one active (controlling) and one passive (monitoring) flow sensor were connected per chip. Specifically, two flow sensors (FS1 and FS3, Figure 1A) were used to control the flow rate via feedback to the pressure controller (i.e., deviation from the set value actuated a pressure change; Figure 3). Two additional flow sensors (FS2 and FS4, Figure 1A) were used to monitor the flow rate, with no feedback to the pressure controller (i.e., deviation from set value did not actuate a pressure change). The configuration used in this setup ensured that the flow rate monitored by FS2 was controlled by feedback from FS3 for SDS addition (i.e., where the last common branch point was either the manifold after the MUX distribution or the pressure inlet to Reservoirs 4/5, depending on valve configuration). Similarly, the flow rate monitored by FS4 was controlled by feedback from FS1 (the last common branch point was the cross‐junction after Reservoir 6 or the pressure inlet to Reservoirs 7/8) or feedback from FS3 (the last common branch point being the manifold after the MUX distribution).

As flow instabilities can arise from passive flow splitting, resistance must be added. Flow rate (Q) is proportional to the pressure drop ( ∆P = P_inlet_ − P_outlet_) through the system and inversely proportional to the resistance (R) of the circuit (Equation 1).

(1) $$Q=\frac{∆P}{R},$$

where ΔP is the pressure difference between the reservoir and each outlet, Q is the flow rate, and R is the resistance.

To ensure that the flow rate was uniform in all the channels (600 μL/min),^18^ an equal length of resistance tubing was added to each line (rigid tubing internal diameter, 300 μm) and a feedback loop between the flow sensor and pressure controller was tuned to achieve a stable flow rate (proportional–integral–derivative [PID] control; Figure 3).

**FIGURE 4:**
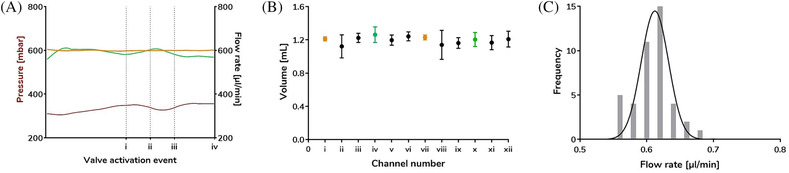
Flow rate analysis of 12 simultaneously perfused channels. (A) Recorded flow rate (orange, FS1 controlling, Channel i; green, FS2 monitoring, Channel iii) and pressure (red line, FS1) during one experiment (42 min). Activation of the valves at different injection time points (i: 20 min, ii: 26 min, iii: 32 min, iv: 42 min) did not affect pressure or flow rate. (B) Volume collected for 2 min for each channel (mean ± standard deviation [SD] of four collection points, t = 16, 22, 28, 36 min; orange dots, FS1 and FS3 controlling; green dots, FS2 and FS4, monitoring; black dots, channels with no flow sensor before the channel inlet). (C) Frequency distribution of data from panel B, expressed as flow rate (bin width 0.02). Gaussian distribution *R* ^2^ = 0.84.

The uniformity of the flow rate in all 12 channels was tested under release assay experimental conditions in the absence of cells. First, the pressure and flow rate were recorded for both pairs of flow sensors per chip, for example, FS1 (FS3) controlling and FS2 (FS4) monitoring. The flow rate of FS1 (controlling) was stable at 599.2 ± 4.7 μL/min over 42 min. The pressure needed to maintain the set flow rate did not show any sudden variation throughout the experiment (335.4 ± 17.6 mbar), indicating optimal resistance (Figure 4A). Similarly, the presence of optimal resistance ensured that the flow rate recorded by FS2 (monitoring) remained within 98.7% of FS1 (i.e., 591.2 ± 14.8 μL/min) (Figure 4A). Uniform flow rate profiles were similarly demonstrated for FS3/4 (data not shown).

The volume at the outlet of all channels was collected for 2 min at representative time points (t = 16, 22, 28, 36 min) to assess the precision of the flow rate and fraction collector (Figure 4B). Volumes collected from the two channels controlled by flow sensors FS1 and FS3 displayed only 1.1%–2.5% variation from the expected output of 1.2 mL (i.e., 600 μL/min × 2 min) at 1.213 mL (FS1) and 1.230 mL (FS3). The volume collected from the two channels monitored by flow sensors FS2 and FS4 remained within 95% of the set value. The mean flow rate of all channels with no flow sensor before the inlet was 587.5 ± 53.3 μL/min, within 97.9% of the set flow rate, with no significant difference from the set value (*p* > 0.05, one‐way ANOVA). The frequency distribution of all volumes collected followed a Gaussian distribution, with a mean of 596.3 ± 48.5 μL/min, supporting the reproducibility of the flow system (Figure 4C). The analysis was repeated in the presence of cells yielding a similar result, that is, controlled channels and those without feedback control did not differ significantly from the 600 μL/min set value of FS1 and FS3 (*p* > 0.05).

### 3.3 Evaluation of monoamine release initiated by test compounds

**FIGURE 5:**
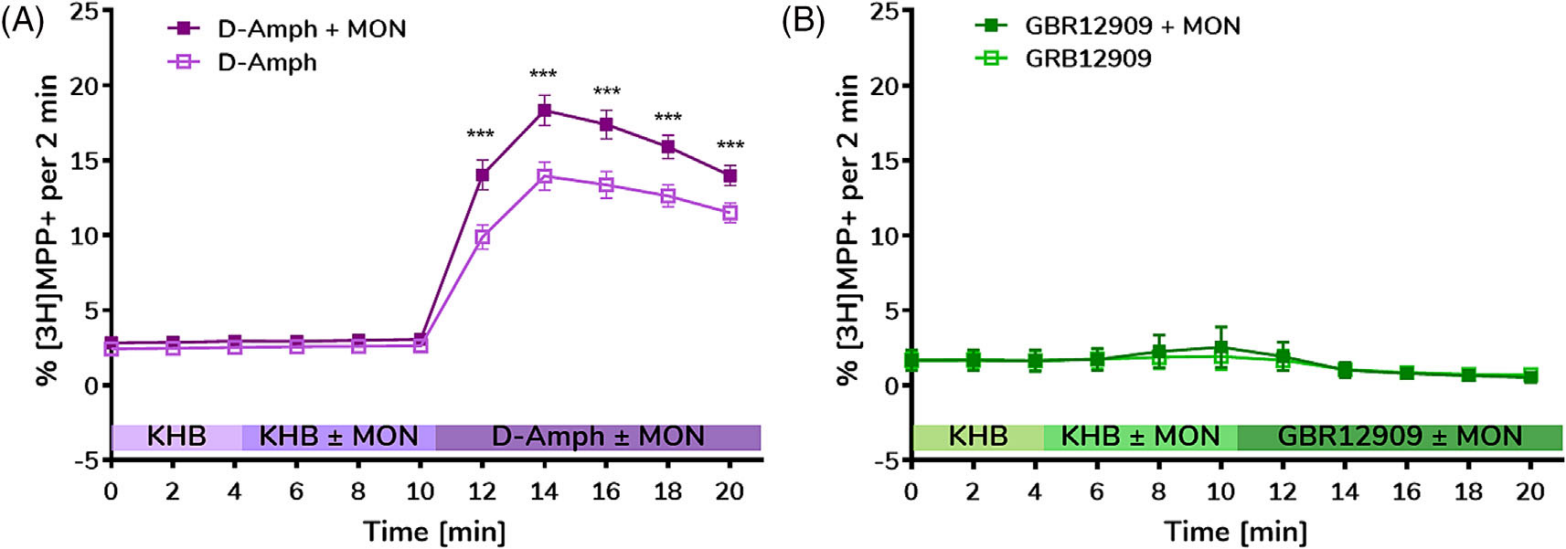
Release assay for human norepinephrine transporter (hNET) using a microfluidic perfusion platform. Parallelized study of the effects of (A) D‐Amphetamine (D‐Amph), (B) GBR12909 with and without monensin on transporter‐mediated release of preloaded radiolabeled substrate [^3^H]MPP+ from human embryonic kidney (HEK293) cells stably expressing hNET. After 6 min of monensin (MON) or Krebs–Henseleit buffer (KHB), the compound of interest ± MON was added. Release of radiolabeled substrate was detected for D‐Amph while no release was observed for GBR12909. A statistical analysis with two‐way ANOVA model, employing Sidak's test for multiple comparison, confirmed significant differences between D‐Amph ± MON at the indicated time points. *p*‐values D‐Amph: *p* < 0.001, 12 min ≤ t ≤ 20; GBR12909: t = 12 min *p* = 0.8912, t = 14 min *p* > 0.9999, t = 16 min *p* > 0.9999, t = 18 min *p* > 0.9999, t = 20 min *p* = 0.9958). Data are presented as percent of released tritium per fraction compared to the radioactivity remaining in the cells at the end of the previous fraction (mean ± standard deviation [SD]).

Once the consistency of the flow rate was established, the release assay was performed with control compounds D‐Amph, GBR12909 with hNET, and paroxetine, pCA with hSERT, to validate the system as an automated procedure for cell‐based assays. D‐Amph was added to HEK293 cells stably expressing the NE transporter (hNET). As expected, an increase in efflux was observed when the compound was injected (t = 10 min), with maximal 14% efflux reached at t = 12 min (Figure 5A). The presence of MON augmented D‐Amph‐caused release, which reached a maximum of 17% and differed significantly (*p* = 0.023) from sole exposure to D‐Amph, consistent with the known effect of D‐Amph as a releasing agent.^21^, ^52^, ^53^ Following the same protocol, GBR12909 was tested as an inhibitor of hNET. As expected, no increase in efflux was observed when the compound was injected (t = 10 min) and no difference was observed in the presence of MON (*p* > 0.05) (Figure 5B). The ability of the microfluidic platform to accurately profile releasing/inhibitory properties of compounds on hNET strongly supports the suitability of the platform for performing these assays.

**FIGURE 6:**
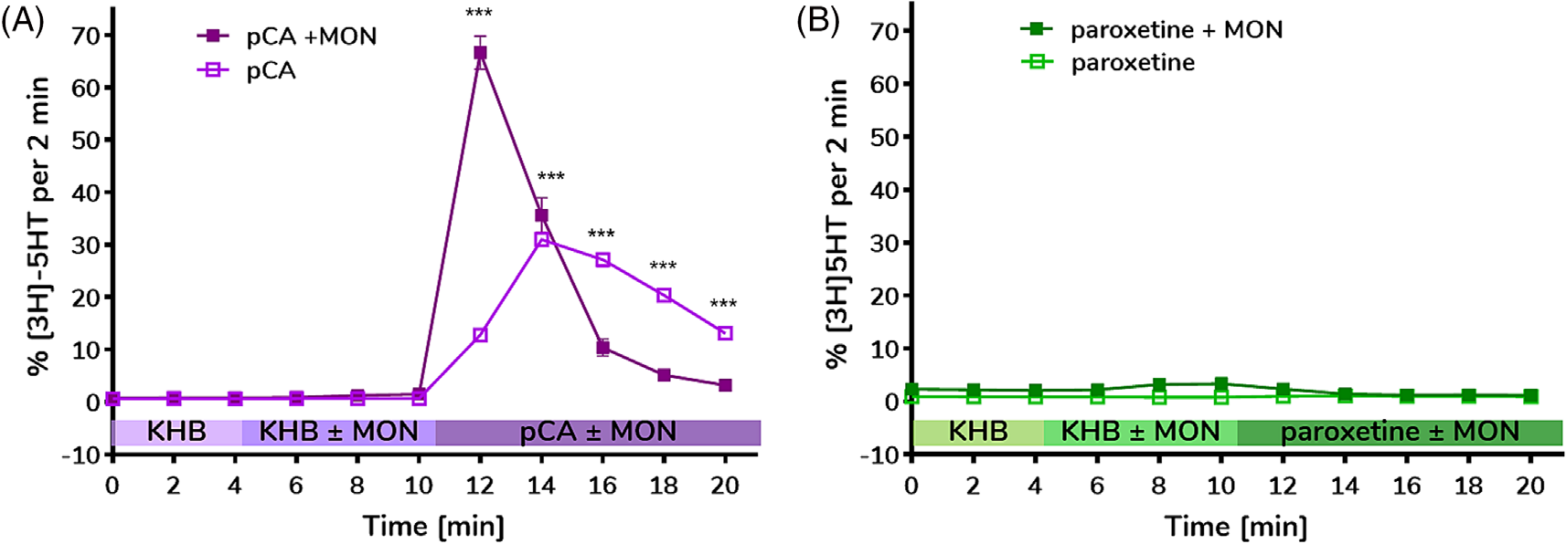
Release assay for human serotonin transporter (hSERT) using a microfluidic perfusion platform. Parallelized study of the effects of (A) p‐chloroamphetamine (pCA) and (B) paroxetine with and without monensin on transporter‐mediated release of preloaded radiolabeled substrate [^3^H]5‐HT from human embryonic kidney (HEK293) cells stably expressing hSERT. After 6 min of monensin (MON) or Krebs–Henseleit buffer (KHB), the compound of interest ± MON was added. Release of radiolabeled substrate was detected with *p*CA and not with paroxetine. A statistical analysis with two‐way ANOVA model, employing Sidak's test for multiple comparison, confirmed significant differences between pCA ± MON at the indicated time points. *p*‐values, *p*CA: *p* < 0.001, 12 min < t ≤ 20 min, paroxetine: t = 14 min *p* < 0.2503, t = 16 min *p* > 0.9999, t = 18 min *p* = 0.9983, t = 20 min *p* = 0.9992). All experiments were conducted three separate times, in triplicate. Data are presented as percent of released tritium per fraction compared to the radioactivity remaining in the cells at the end of the previous fraction (mean ± standard deviation [SD]).

The microfluidic platform was additionally tested with hSERT. pCA was added following the release assay protocol. *p*CA potently induced a maximum efflux of 30% (t = 14 min), while a significantly increased release of 67% was observed in the presence of MON (t = 12 min) (Figure 6A). For the hSERT‐inhibiting compound, paroxetine, efflux was not observed in the presence or absence of MON (*p* > 0.05), consistent with previous studies^21^ (Figure 6B). Taken together, the low variability for each time point and stable baseline efflux demonstrate the robustness of the microfluidic perfusion platform for efflux assays.

## 4 DISCUSSION

The present study describes the development of a new microfluidic setup suitable for automating multi‐step cell‐based assays, demonstrated here for release assays. While the addition of microfluidic flow control introduces benefits of physical and temporal relevance over static in vitro models, fluidic cell‐based assays ranging in complexity from 2D assays to 3D models and organ‐on‐chip technology currently suffer from a general lack of standardization.^54^ Many custom devices are reported and components such as pumps and microfluidic chips are often built in‐house.^46^, ^47^, ^55^ Not only does this make experimental conditions difficult to reproduce, but results can be difficult to compare without an expert understanding of the physical concepts of fluid behaviour. In addition, existing methods largely require manual operation of complex and/or repetitive steps, leading to challenges with reproducibility and interoperability. The key advance of our technology for the field of in vitro cell‐based assays is the ability to automate a series of multiple injection sequences to simultaneously perfuse different solutions into up to 12 different channels with the precision afforded by a pressure‐driven flow controller and flow sensor feedback loops. The suitability of our microfluidic platform for the standardization of in vitro cell‐based assays is due to a combination of features, specifically: (1) The use of instruments that meet industry‐level precision and accuracy, presenting an improvement over the commonly used syringe and peristaltic pumps^56^; (2) the ability to tune and automate parameters such as flow rate and volume control and programme sequential injections to reduce manual handling errors and time burden for the researcher.^47^ Indeed, measurement of microfluidic parameters is recognized as a priority target for standardization of in vitro cell assays^54^; (3) the simultaneous control of triplicates of four experimental conditions to address the demand for parallelization; (4) compatibility with commercially available microfluidic chips that have standard geometries, and thus, known and reproducible flow and shear stress profiles^57^; (5) compatibility with existing readout technologies, including optical microscopy of cells in the microfluidic chip and off‐chip analysis of collected samples; and (6) the added ecological and economic benefits of reduced system volume, resulting in up to 50% less reagents used and waste produced.^36^ While the use of radioactive labels allows tracer flux assays to be performed in a highly sensitive manner, it carries certain disadvantages such as the use of radioactivity per se, relatively high costs and complicated workflows due to the caution necessary for the handling of such compounds. Of note, a fluorescence‐based sensor^58^, ^59^ was recently developed to measure DA uptake and release through the DA transporter, allowing the detection of neurotransmitters without the need for radioactivity. The potential merging of this assay with our demonstrated platform presents an exciting future opportunity to couple microfluidic automation and enhanced precision with improved assay safety and even greater cost savings. Together, by supporting the standardization of instruments, components and protocol automation, our platform represents a step towards the standardization of in vitro cell‐based assays and further reduces barriers to the adoption of the technology by non‐specialists in microfluidics.^60^

Parallelization has come to be increasingly necessary with the rapid evolution of compound libraries and chemical synthesis techniques to accelerate the screening of new substances.^61^ While cell‐based assays for such high content screening typically target 96 or 384‐well plates or microarray setups,^62^, ^63^ here, we prioritized the automation of a multi‐sequential injection protocol to study monoamine transporter‐mediated substrate efflux in triplicates of four simultaneous conditions following an existing assay. In order to minimize the equipment needed, while preserving the option to scale up the system and increase parallelization, only two of the four available air outlets of the pressure‐driven flow controller were used in this setup. In this configuration, the flow rate of six channels was dependent on the feedback of one flow sensor. To ensure high stability and precision of flow control for a uniform flow rate in each channel, each line was kept the same length and equipped with high‐resistance tubing. Typically, resistance is a key component to stabilize microfluidic flow setups that present a series of junctions.^64^ The symmetry of our microfluidic design, where FS1 and FS3 each controlled flow through half of each chip, also contributed to the low flow rate variability between conditions and triplicates. Therefore, it is reasonable to assume that the throughput of this setup, using one pressure controller, could be parallelized further to double the number of assays performed simultaneously to 24 (i.e., using four chips of six channels each).

In order to validate the system, the well‐characterized effects of selected compounds were benchmarked to compare the novel platform to existing systems. Qualitatively, our platform reproduced the expected efflux profiles very well. The differences observed in maximum efflux and efflux rate are likely explained by differences in chip geometry. With the addition of *p*CA, our platform showed a max [^3^H]5‐HT efflux from hSERT that was 1.5‐fold higher than reported by Scholze et al. and Mayer et al.^21^, ^50^ and a sharp decrease after initial maximum release, compared to a more sustained release in the previous studies. The shape and dimensions of the microfluidic chip used in our platform generated a laminar flow with a parabolic flow profile, while perfusion chambers^21^, ^65^ produce vortexes in the space surrounding the cells due to the presence of an obstacle (i.e., glass coverslip).^66^ It is possible that the laminar flow profile increased the clearance rate of the substrate compared to the more perturbed flow present in the perfusion chamber. Faster clearance would decrease the residence time of the substrate in close proximity to the cells and NTTs, therefore decreasing the chances of reuptake (for subsequent re‐release) and increasing the initial amount of substrate collected at the outlet of the chip. Thus, cells would quickly be depleted of radioactive substrate, explaining the low fractional release for the condition pCA + MON in our platform only 4 min after maximum release, compared to other studies.^21^, ^50^

In summary, a microfluidic platform was successfully developed for the simultaneous perfusion of 12 channels using up to eight different solutions. Automation enabled the establishment of a more standardized protocol for the release assay, while also satisfying the demand for precise and stable flow control, and reducing the need for time‐consuming manual operation, thereby decreasing the likelihood of operator errors. The user‐friendly platform was optimized to automate release assays, however, it can be adapted to other cell‐based assays that include perfusion and/or simultaneous sequential injection of one or more solutions, such as immunoscreening assays and functional assays based on a biomarker readout.^67^ The automation, along with time reduction and the availability of commercial accessories, opens up the use of microfluidics to a broader range of researchers and represents a step closer to standardization.

## CONFLICT OF INTEREST STATEMENT

At the time of this work, F.R.B., C.I., L.D.M. and B.G. were employees of Elvesys SAS, a for‐profit company that sells Elveflow equipment, which was used for flow control and measurements in this work.
